# Key risk factors of generalized anxiety disorder in adolescents: machine learning study

**DOI:** 10.3389/fpubh.2024.1504739

**Published:** 2025-01-07

**Authors:** Yonghwan Moon, Hyekyung Woo

**Affiliations:** ^1^Department of Health Administration, Kongju National University of Nursing and Health, Kongju, Republic of Korea; ^2^Institute of Health and Environment of Kongju National University, Kongju, Republic of Korea

**Keywords:** adolescent, mental health, generalized anxiety disorder, machine learning, health behaviors

## Abstract

Adolescents worldwide are increasingly affected by mental health disorders, with anxiety disorders, including Generalized Anxiety Disorder (GAD), being particularly prevalent. Despite its significant impact, GAD in adolescents often remains underdiagnosed due to vague symptoms and delayed medical attention, highlighting the need for early diagnosis and prevention strategies. This study utilized data from the Korea Youth Risk Behavior Web-based Survey (KYRBS) from 2020 to 2023 to analyze factors influencing GAD in adolescents. Using machine learning techniques such as Lasso Regression, SelectKBest, and XGBoost, we identified key variables, including health behaviors such as sleep, smoking, and fast-food intake, as significant factors associated with GAD. Predictive models using Random Forest and Artificial Neural Networks demonstrated that the XGBoost feature selection method effectively identified key factors and showed strong performance. These findings emphasize the need for educational programs focusing on sleep management, smoking prevention, and balanced nutrition to reduce the risk of GAD in adolescents, providing crucial insights for early diagnosis and intervention efforts.

## 1 Introduction

According to mental health data from the World Health Organization (WHO), 14% of adolescents worldwide experience mental disorders (1). Inadequate management of these disorders can lead to severe disruptions in social functioning, with serious consequences, such as suicide (2). Among mental disorders, anxiety disorders are particularly prevalent, affecting an estimated 3.6% of the individuals aged 10–14 years and 4.6% of those aged 15–19 years (1), a higher prevalence compared to attention deficit hyperactivity disorder and depression (3). Generalized Anxiety Disorder (GAD), a subtype of anxiety disorders, often manifests during adolescence and is characterized by persistent worry and anxiety in daily life (4). According to the Diagnostic and Statistical Manual of Mental Disorders, Fifth Edition, GAD is diagnosed when excessive anxiety or worry persists for at least 6 months, is difficult to control, and is accompanied by at least three symptoms, including restlessness, fatigue, difficulty concentrating, irritability, muscle tension, and sleep disturbances (5).

Despite the increasing prevalence of GAD among adolescents, it remains relatively uncommon for individuals to seek medical attention specifically for anxiety, often only doing so after the condition has become chronic or presents with vague symptoms (6). The nature of GAD is often perceived as introverted, which may delay early detection compared to other mental disorders (7). Adolescence is a critical developmental stage marked by psychological and physical changes, which makes individuals particularly vulnerable. A delayed diagnosis of GAD can significantly increase the risk of school refusal, substance abuse, and reduced quality of life (6, 8). If mental health issues such as GAD are not properly managed during adolescence, they can hinder healthy growth and development throughout life, highlighting the importance of early diagnosis and intervention to prevent chronicity (9).

Several factors influencing GAD in adolescents have been reported, including sex, socioeconomic status, stress, and subjective health perceptions (7). Sociodemographic characteristics, health behaviors, and health perceptions, such as stress, feelings of hopelessness, subjective health status, and recovery from fatigue after sleep, have been analyzed in relation to GAD (10). In addition, studies have explored the association between secondhand smoke exposure and GAD (11), as well as the impact of habitual substance use (12). While ongoing research continues to explore these associations, previous data may not fully capture changes in adolescent health behaviors due to evolving living environments. Therefore, a focused examination of adolescent health behaviors is necessary.

Recently, machine learning (ML) has emerged as a transformative tool in mental health research, utilizing large-scale datasets to develop predictive models that go beyond human recognition. These models are effective not only in identifying known variables but also in discovering complex patterns, showing superior accuracy over traditional methods. For instance, ML has differentiated between anxiety subtypes like generalized anxiety disorder (GAD) and social anxiety disorder (SAD) using functional magnetic resonance imaging data with accuracy up to 94% (13). Additionally, it has been applied to predict anxiety and depression based on sociodemographic, lifestyle, and nutritional data, uncovering key predictors such as potassium and vitamin E intake (14, 15). ML has also been used to predict treatment outcomes, such as cognitive behavioral therapy success (16). However, the application of ML in understanding GAD-specific risk factors, especially in adolescents, remains underexplored. This study seeks to address this gap, emphasizing the need for systematic comparisons of ML algorithms to identify optimal approaches for risk prediction and intervention strategies tailored to adolescents.

This study investigated the factors associated with GAD in adolescents using data from the Korea Youth Risk Behavior Web-based Survey (KYRBS) from 2020 to 2023, during which time GAD variables were first introduced. By employing various machine learning algorithms to analyze the data, we sought to provide insights for the development of early diagnosis, treatment, and prevention programs for adolescent GAD.

## 2 Methods

### 2.1 Study population

We utilized raw data from the 16th to 19th KYRBS. The KYRBS is a self-administered online survey conducted annually by the Korea Disease Control and Prevention Agency since 2005. It involves 800 sample schools nationwide (400 middle schools and 400 high schools), targeting students aged 12–18 years, from the first year of middle school to the third year of high school. For this study, from the total 214,344 participants in the KYRBS conducted from 2020 to 2023, 213,820 participants were selected for final analysis, excluding 524 individuals who reported an average sleep time of 0 h over the past 7 days.

### 2.2 Study variables

The dependent variable in this study was GAD, while independent variables included sociodemographic characteristics (8 items: sex, grade, subjective economic status, subjective academic performance, living situation, family composition, parental education level, and parental nationality), physical health characteristics (6 items: body mass index, subjective health status, subjective body shape status, number of days engaged in strength training, number of days participating in high-intensity physical activity, and sitting time), lifestyle characteristics (5 items: sleep quality, smartphone usage time, and fatigue recovery from sleep), dietary habits (5 items: frequency of sugary drink intake, consumption of fast food, fruit intake, breakfast intake, and water intake), violence and deviant behavior (3 items: alcohol intake, smoking experience, and treatment for violence-related injuries), disease characteristics (3 items: asthma, allergic rhinitis, and atopic dermatitis), mental health characteristics (2 items: stress and feelings of loneliness), and sexual behavior (1 item: experience of sexual intercourse).

### 2.3 GAD

GAD was measured using the Generalized Anxiety Disorder-7 (GAD-7) tool. The GAD-7 questionnaire asked participants: “Over the last 2 weeks, how often have you been bothered by the following problems?” The seven items included: “Feeling nervous, anxious, or on edge,” “Not being able to stop or control worrying,” “Worrying too much about different things,” “Having trouble relaxing,” “Being so restless that it is hard to sit still,” “Becoming easily annoyed or irritable,” and “Feeling afraid as if something awful might happen.” Responses were rated on a Likert scale from 0 (not at all) to 3 (nearly every day), with total scores ranging from 0 to 21. Higher scores indicated a greater GAD severity. Following established research on the development of the GAD-7 (17), a score ≥ 10 classified participants as being in the high-risk group for GAD, while a score < 10 classified them as low risk.

### 2.4 Study model and analysis method

**Figure 1:**
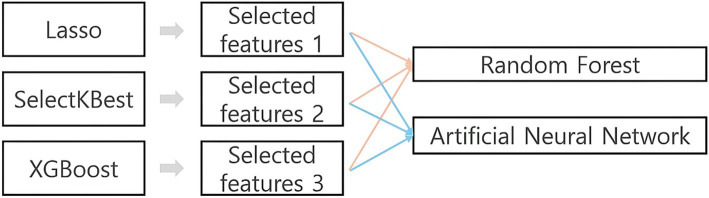
Study model.

A research model was established to compare selected features and model performance for each algorithm (Figure 1). Analysis was conducted using Python 3.12.2. To address missing data, the K-Nearest Neighbors algorithm was applied. This method identifies the nearest K neighbors based on available values and replaces missing values with the mean or median of these neighbors, reducing bias in the estimated values compared to regression imputation or mean imputation methods (18). To ensure comparability among variables, all independent variables were standardized using z-score transformation before being included in feature selection and prediction models. Standardization scaled each variable to have a mean of 0 and a standard deviation of 1, ensuring consistent input for all algorithms. To identify factors influencing GAD, feature selection was performed using least absolute shrinkage and selection operator (LASSO) regression, SelectKBest, and XGBoost. Frequency analysis was conducted to understand the general characteristics of the study participants, and Random Forest (RF) and Artificial Neural Network (ANN) models were built using the selected features to evaluate prediction performance.

### 2.5 SMOTE algorithms

SMOTE was applied to address the class imbalance problem. SMOTE is an algorithm that improves data imbalance and enhances the model’s ability to learn from the minority class by generating synthetic samples for the underrepresented class (19). However, SMOTE may lead to overfitting due to the introduction of synthetic samples. To address this potential drawback, we evaluated performance metrics such as sensitivity and precision, and employed regularization methods like Lasso and ensemble techniques such as Random Forest to mitigate overfitting risks (20).

### 2.6 Feature-selection algorithms

We selected LASSO, SelectKBest, and XGBoost as feature selection methods due to their proven effectiveness and alignment with the goals of our analysis. LASSO was chosen for its strength in addressing multicollinearity while simplifying the model by reducing less relevant feature coefficients to zero, making it highly interpretable (21). SelectKBest was used to prioritize features that exhibited the strongest statistical correlation with the target variable, ensuring the model concentrated on the most impactful variables (22). XGBoost, with its robust ensemble learning framework, was particularly advantageous for identifying non-linear relationships and interactions among features (23). While alternative methods such as Recursive Feature Elimination and mutual information-based selection were considered, they were deemed less suitable due to their computational inefficiency and limitations when applied to high-dimensional datasets (24, 25). The chosen methods not only optimized model performance but also supported a clear interpretation of the variables influencing GAD in adolescents.

### 2.7 Model algorithms

Two model algorithms, Random Forest (RF) and Artificial Neural Network (ANN), were utilized in this study. RF is an ensemble model designed to mitigate overfitting and generalization issues in traditional decision trees by constructing multiple decision trees to enhance predictive performance (26). ANN consists of an input layer, hidden layers, and an output layer, with nodes connected through weights and activation functions, enabling accurate pattern recognition and prediction (27). Hyperparameters for both models were optimized using grid search. For RF, the best parameters were: max_depth = None, max_features = “sqrt,” min_samples_leaf = 2, min_samples_split = 10, and n_estimators = 200 (LASSO); max_depth = None, max_features = “log2,” min_samples_leaf = 1, min_samples_split = 5, and n_estimators = 100 (SelectKBest); and max_depth = 10, max_features = “log2,” min_samples_leaf = 1, min_samples_split = 2, and n_estimators = 100 (XGBoost). For ANN, the optimal settings included: batch_size = 32, epochs = 20, learning_rate = 0.01, and units = 32 (LASSO); batch_size = 64, dropout_rate = 0.3, epochs = 20, neurons1 = 64, neurons2 = 16, and optimizer = “rmsprop” (SelectKBest); and batch_size = 16, epochs = 20, neurons1 = 32, neurons2 = 16, and optimizer = “adam” (XGBoost).

## 3 Results

### 3.1 Feature selection

The feature selection results are presented in Table 1. Variables commonly selected across all algorithms included loneliness, stress, inadequate recovery from sleep-related fatigue, poor subjective health perception, experience of treatment due to violence, low subjective economic status, insufficient weekend sleep, and smoking experience. Among these, loneliness emerged as the most significant variable with the highest absolute coefficient value (0.23) in LASSO, the highest F-score (16,646) in SelectKBest, and the highest gain (0.29) in XGBoost.

**Table 1:** Feature selection results.

	Variable importance		
Variables	LASSO (absolute coefficient value)	SelectKBest (F-score)	XGBoost (Gain)
Loneliness	0.23	16,646	0.29
Stress	0.1	6,589	0.07
Poor subjective health perception	0.12	6,782	0.04
Inadequate fatigue recovery from sleep	0.1	5,081	0.02
Experience of treatment due to violence	0.1	5,117	0.01
Poor subjective economic status	0.03	1,381	0.01
Insufficient weekend sleep	0.03	1,003	0.01
Smoking	0.03	643	0.01
Sexual experience	0.2	11,219	–
Water intake (less than once daily)	0.02	–	–
Allergic rhinitis	–	982	–
Sex (female)	–	–	0.004
Fast food intake (daily)	–	–	0.02

In addition, sexual experience was selected by all algorithms except XGBoost, with a significant absolute coefficient value (0.2) in LASSO and an F-score of 11,219 in SelectKBest. Other variables, such as water intake of less than once daily (absolute coefficient value of 0.02 in LASSO), allergic rhinitis (F-score of 982 in SelectKBest), female sex (gain of 0.004 in XGBoost), and daily fast food intake (gain of 0.02 in XGBoost), were also identified as important features, though they were not consistently selected across all algorithms.

### 3.2 General characteristics of the study population

The general characteristics are presented in Table 2. The sex distribution was skewed toward males (61.8%). In terms of school year, 53.8% of the participants were middle school students, slightly outnumbering high school students. The majority of participants rated their subjective economic status as “middle” (47.4%), while the most common rating for subjective academic performance was “high” (37.5%). Most of the students lived with their families (95.2%), and both fathers and mothers predominantly had a university education (44.4 and 44.2%, respectively). Regarding subjective health status, 65.7% of the participants rated their health as “high,” while 25.1% reported it as “middle,” and 9.3% as “low.” Recovery from sleep-related fatigue was reported as “insufficient” by 41.1% of the participants. The frequency of consumption of fast food was highest at 1–2 times per week (56.9%), and 38.2% of the participants reported drinking more than five cups of water daily. Alcohol consumption was reported by 33.2% of the participants, smoking by 9.3, and 9.4% had sought treatment due to violence. Allergic rhinitis was diagnosed in 41.6% of the participants. Notably, 81.2% reported experiencing high levels of stress, 52.6% experienced loneliness, and 27% reported having had sexual intercourse. The prevalence of GAD in the study population was 17.9%.

**Table 2:** General characteristics (*n* = 213,820).

Variables	Category	*n* (%)
Sex	Male	132,211 (61.8)
	Female	81,609 (38.2)
Grade	Middle school	114,966 (53.8)
	High school	98,854 (46.2)
Subjective economic status	High	25,603 (12.0)
	Medium	101,295 (47.4)
	Low	86,922 (40.7)
Subjective academic performance	High	80,466 (37.5)
	Medium	64,331 (30.0)
	Low	69,015 (32.2)
Living situation	Living with family	204,146 (95.2)
	Other	10,194 (4.8)
Father’s education	Below middle school	2,225 (1.0)
	High school graduate	34,508 (16.2)
	College graduate or higher	95,029 (44.4)
	Unknown	82,058 (38.4)
Mother’s education	Below middle school	1,789 (0.8)
	High school graduate	39,699 (18.6)
	College graduate or higher	94,538 (44.2)
	Unknown	77,794 (36.4)
Subjective health status	High	140,428 (65.7)
	Medium	53,570 (25.1)
	Low	19,822 (9.3)
Fatigue recovery from sleep	Adequate	55,090 (25.8)
	Average	70,809 (33.1)
	Inadequate	87,921 (41.1)
Fast food intake	None in the last 7 Days	36,565 (17.1)
	1–2 times a week	121,576 (56.9)
	3–4 times a week	44,273 (20.7)
	5–6 times a week	7,461 (03.5)
	Daily 1 times	2,546 (1.2)
	Daily 2 times	674 (0.3)
	Daily 3 times	725 (0.3)
Water intake	Less than 1 cup daily	7,989 (3.7)
	1–2 cups daily	38,912 (18.2)
	3 cups daily	47,480 (22.2)
	4 cups daily	37,764 (17.7)
	5 or more cups daily	81,675 (38.2)
Smoking	Smoker	19,985 (90.7)
	Non-smoker	193,835 (9.3)
Experience of treatment due to violence	Yes	20,035 (9.4)
	No	193,785 (90.6)
Lifetime diagnosis of allergic rhinitis	Yes	88,989 (41.6)
	No	124,831 (58.4)
Stress	Yes	173,975 (81.2)
	No	40,365 (18.8)
Loneliness experience	Yes	112,367 (52.6)
	No	101,453 (47.4)
Generalized anxiety disorder	High-risk group	38,288 (17.9)
	Low-risk group	175,532 (82.1)

### 3.3 Analysis

**Figure 2:**
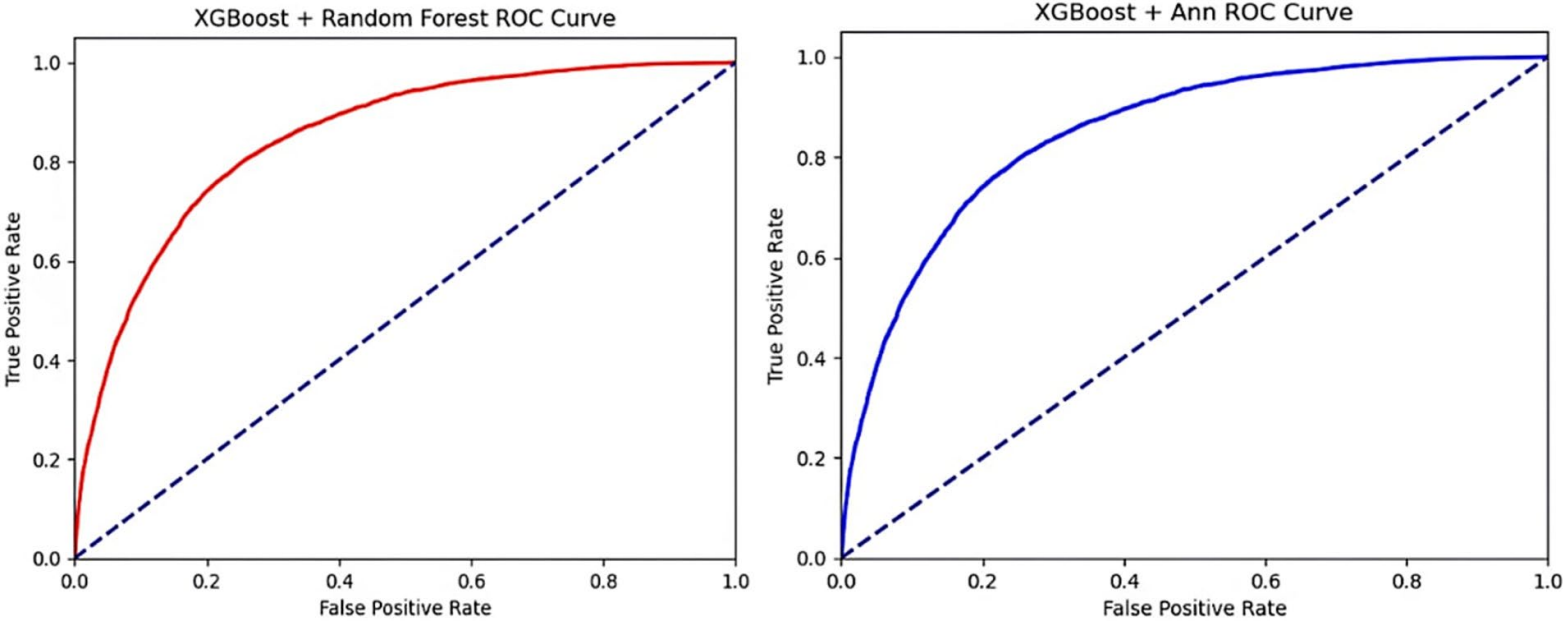
**(A)** XGBoost + Random Forest ROC Curve. **(B)** XGBoost + Ann ROC Curve.

The performance results of the RF and ANN models using selected features are shown in Table 3 and Figure 2. For the RF model, the XGBoost feature-selection method demonstrated accuracy of 78% ± 0.0011, AUC of 82% ± 0.0004, sensitivity of 64% ± 0.0026, precision of 43% ± 0.0017, and specificity of 81% ± 0.0017. The higher sensitivity of XGBoost may be attributed to its ability to capture complex feature interactions and reduce overfitting through gradient-boosted decision trees. In contrast, SelectKBest achieved the highest accuracy (81% ± 0.0003) and specificity (90% ± 0.0007), making it ideal for scenarios where minimizing false positives is critical.

**Table 3:** Model performance.

	Lasso		SelectKBest		XGBoost	
	%	SE	%	SE	%	SE
**Random forest**						
Accuracy	79	0.0008	81	0.0003	78	0.0011
AUC	35	0.0009	78	0.0003	82	0.0004
Sensitivity	72	0.0006	39	0.0006	64	0.0026
Precision	40	0.0007	46	0.0007	43	0.0017
Specificity	88	0.0006	90	0.0007	81	0.0017
**ANN**						
Accuracy	80	0.0006	82	0.0005	80	0.0021
AUC	74	0.0006	81	0.0004	81	0.0012
Sensitivity	31	0.0053	44	0.0079	56	0.0043
Precision	41	0.0055	51	0.0039	45	0.0038
Specificity	90	0.0060	90	0.0072	85	0.0032

SE, Standard error.

For the ANN model, XGBoost continued to excel with a sensitivity of 56% ± 0.0043 and AUC of 81% ± 0.0012, reflecting its robustness in handling nonlinear relationships. Meanwhile, SelectKBest achieved balanced performance with accuracy (82% ± 0.0005) and specificity (90% ± 0.0072), while LASSO offered slightly lower predictive performance but added value in reducing model complexity by selecting key features in high-dimensional datasets.

Overall, the standard errors for all algorithms were low, indicating stable performance across iterations. However, the superior sensitivity of XGBoost suggests it is particularly suitable for tasks emphasizing the detection of generalized anxiety disorder, where underdiagnosis must be minimized.

## 4 Discussion and conclusion

We investigated the factors influencing GAD in adolescents by analyzing data from the KYRBS from 2020 to 2023. Various machine learning techniques, including the LASSO, SelectKBest, and XGBoost feature-selection algorithms, were used to explore and extract key variables. The findings highlight that adolescent GAD is closely associated with health behaviors, reinforcing insights previously documented in the literature. Based on the selected variables, RF and ANN models were constructed to evaluate predictive performance. The evaluation demonstrated similar accuracy and AUCs across the methods but XGBoost feature selection exhibited the highest sensitivity. Among the three feature-selection methods, XGBoost exhibited superior overall performance in both models, emphasizing its robustness in identifying influential factors. Several important points arise from these results.

First, the association between loneliness and GAD aligns with previous studies, which have shown that loneliness among adolescents contributes to increased anxiety levels (28). Adolescence is a critical period for forming social relationships, and the experience of loneliness during this stage can lead to feelings of despair and helplessness, potentially fostering anxiety (29). Loneliness can disrupt emotional stability and increase the long-term risk of GAD. Similarly, stress emerged as another major contributing factor, corroborating earlier findings that stress, particularly related to school life, increases anxiety (7). Stress from peer relationships, academic pressures, and perceived discrimination from teachers was found to significantly elevate the risk of GAD (30). Unlike many prior studies that predominantly utilized linear models, this research employed both linear (e.g., Lasso) and non-linear (e.g., XGBoost) approaches to validate the significance of loneliness and stress as predictors of GAD. By incorporating non-linear models like XGBoost, this study was able to identify complex interactions and relationships between variables that may have been overlooked in earlier studies relying solely on linear models. The findings demonstrate that the effects of loneliness and stress on GAD are robust across different analytical methods, suggesting that both linear and non-linear relationships play a crucial role in adolescent anxiety. This mixed modeling approach provides a more comprehensive understanding of how social factors, such as loneliness and stress, interact to impact adolescent mental health, emphasizing the importance of these variables regardless of the modeling technique used.

Furthermore, experiences of violence were shown to influence the development of GAD. Adolescents who have experienced violence often suffer from both physical trauma and mental health challenges, with previous studies confirming a strong association between violence victimization and increased anxiety (31, 32). Such adolescents may also display emotional instability, manifesting in behaviors such as aggression toward others (33). Consequently, emotional support programs are essential for adolescents who have been victims of violence to mitigate the psychological damage and prevent the development of GAD.

Subjective health perception was another significant factor influencing GAD. Adolescents with a negative perception of their health are more likely to experience mental stress and anxiety (30). This reinforces the importance of promoting positive health perceptions through regular health education programs to support mental and emotional well-being. Similarly, subjective economic status was identified as a contributing factor. Adolescents from lower economic backgrounds are more prone to psychological difficulties, leading to anxious thoughts about their future (34). This highlights the need for psychological counseling for adolescents experiencing economic hardship to help alleviate anxiety.

Differences in the variables selected by each algorithm were noted, with XGBoost selecting female sex and daily intake of fast food as significant predictors. The high risk of anxiety among female adolescents is well-documented, irrespective of their household, economic, or academic conditions (10). This suggests that female students are generally more vulnerable to anxiety, potentially due to psychological and physiological factors. Social pressures, concerns about appearance, and high academic expectations may increase this vulnerability, while hormonal fluctuations, such as changes in estrogen levels, can also influence emotional and behavioral responses, increasing anxiety risk (10, 35). Addressing GAD in female students, therefore, requires interventions tailored to both psychological and physiological factors.

Among the selected features, health behaviors such as recovery from sleep fatigue, weekend sleep, smoking experience, and fast food intake were notably associated with GAD. Previous research on the relationship between sleep quality and anxiety among Korean adolescents has demonstrated that lower sleep quality is correlated with a higher risk of GAD (36). Insufficient weekend sleep further compounds this issue by preventing recovery from weekday sleep deprivation, potentially leading to chronic sleep deficiency, which may result in GAD (37). As such, sleep recovery and weekend sleep play a critical role in managing GAD, highlighting the importance of incorporating sleep-focused strategies into adolescent mental health interventions.

Smoking during adolescence presents more severe physical and mental health risks compared to smoking in adulthood (38). Nicotine exposure at this stage can adversely affect brain regions involved in emotional regulation, such as the prefrontal cortex and limbic system, potentially exacerbating anxiety symptoms (39). It has also been found to disrupt neurotransmitter systems, including dopamine and serotonin pathways, which are crucial for maintaining mental well-being (40). Moreover, adolescents who smoke are more likely to engage in other risky health behaviors, such as poor dietary choices and irregular sleep patterns, which together increase their vulnerability to GAD (41). Similarly, the consumption of fast food, known for its high fat and sugar content, has been linked to poorer mental health outcomes and an increased risk of GAD (42). Conversely, diets rich in essential nutrients have been associated with reduced anxiety symptoms, while diets high in processed foods and refined carbohydrates exacerbate anxiety levels (42). These findings emphasize the need for nutritional education and dietary guidance to promote balanced eating habits. Given that health behaviors, such as sleep, smoking, and diet, are modifiable, effective interventions targeting these areas can significantly reduce the risk of GAD in adolescents.

In addition to exploring the association between health behaviors and GAD, this study utilized feature-selection techniques—LASSO, SelectKBest, and XGBoost—to identify key factors, along with predictive models, including RF and ANN. Comparative studies of machine learning algorithms are increasingly prevalent in mental health research, as applying multiple algorithms allows for more effective handling of unique data characteristics and noise. This flexibility in selecting the most suitable model enhances overall predictive performance (43). In this study, the XGBoost algorithm, a powerful boosting method, exhibited the best performance. XGBoost is known for its ability to prevent overfitting and identify critical variables by considering interactions during the feature-selection process (44). Importantly, the variables selected by XGBoost were consistent with those highlighted in previous studies, indicating that it effectively captured the key factors influencing adolescent GAD. Feature selection using LASSO and SelectKBest was also useful for identifying important variables, and the comparative performance analysis of the RF and ANN models confirmed that machine learning techniques are important for analyzing adolescent GAD. These findings highlight the effectiveness of machine learning in mental health analysis and provide a basis for more refined future research using various algorithms.

Despite its contributions, this study had several limitations. First, the data collection occurred during the COVID-19 pandemic, which may have influenced the results. Second, while the study identified violence as a factor, the results do not differentiate between types or degrees of violence. Future research should categorize violence to provide more detailed insights. Third, although SMOTE was used to enhance the model’s sensitivity and precision, overall performance remained modest. Continuous data accumulation may be necessary to improve the learning of feature patterns associated with GAD. Fourth, the data used in this study was derived from the Korea Youth Risk Behavior Web-based Survey (KYRBS), which relies on self-reported measures that can be prone to biases such as social desirability bias or recall bias. These biases may affect the accuracy of the findings, as reported levels of loneliness, stress, and other health behaviors might be either underreported or overreported, thus influencing the outcomes related to GAD. Despite these limitations, this study is significant in that it used the latest data from 2020 to 2023 to explore various factors influencing GAD in adolescents through machine learning algorithms, providing valuable insights for early intervention and prevention.

Nevertheless, this study holds significant value, as it utilized recent data from 2020 to 2023, marking the first years that included GAD-related items in the Korean Youth Health Behavior Survey. By employing diverse machine learning algorithms, we successfully identified multiple factors influencing GAD. Our results confirm the importance of health behaviors, including sleep recovery, sleep duration, smoking, and consumption of fast food, in GAD risk. These findings suggest the need for integrated educational programs that address these factors, alongside mental health education. In addition, our findings highlight the value of applying various machine learning techniques for feature selection and predictive modeling in research on adolescent GAD. Future research should include longitudinal studies to understand how these factors change over time and integrate physiological or genetic data to better clarify the underlying mechanisms of GAD. Public health strategies focusing on improving sleep hygiene, preventing smoking, and promoting balanced nutrition may effectively reduce the risk of GAD in adolescents.

## Data Availability

Publicly available datasets were analyzed in this study. This data can be found here: https://www.kdca.go.kr/yhs/.
